# Lost in space and time: robust demography and enhanced resilience buffer adverse environmental effects in a highly isolated and sedentary pre-pleistocene landscape vertebrate

**DOI:** 10.1186/s12862-024-02314-2

**Published:** 2024-10-11

**Authors:** Philippe J. R. Kok, Tessa L. Broholm, Loïc van Doorn, Bruno Ferreto Fiorillo, Carl Smith

**Affiliations:** 1 Department of Ecology and Vertebrate Zoology, University of Łódź, 12/16 Banacha Str, Łódź, 90-237 Poland; 2 Department of Life Sciences, The Natural History Museum, London, UK; 3 APHIS Wildlife Services, United States Department of Agriculture, Lihue, HI USA; 4 Research Institute for Nature and Forest - INBO, Brussels, Belgium; 5 Institute of Vertebrate Biology, Czech Academy of Sciences, Brno, Czech Republic

**Keywords:** Animal movement, Capture-mark-recapture, Climate change, Dispersal abilities, Environmental factors, Tepui summit

## Abstract

**Background:**

Few animal populations have been studied under the framework of the OCBIL theory, which addresses the ecology and evolution of biodiversity on old climatically buffered infertile landscapes. Available genetic data challenge the low connectivity and high genetic differentiation predicted for isolated tepui-summit vertebrate communities, suggesting potential dispersal among summits. However, the OCBIL theory posits reduced dispersibility, enhanced resilience to habitat fragmentation and inbreeding due to small populations. We tested these hypotheses by conducting the first analytic evaluation of the spatial ecology and population biology of a tepui-summit vertebrate at multiple spatial scales.

**Results:**

We used harmonic radar tracking (100 individuals/448 points of contact) and capture-mark-recapture data (596 individuals captured/52 recaptured) to reveal the temporal niche, microhabitat use, population size, and dispersal abilities of the tepui-summit endemic toad *Oreophrynella quelchii* on Roraima-tepui. Abundance was determined using a closed population model incorporating sources of variation in capture probability. We tested the relative influence of biotic and abiotic variables on distances moved through model selection. Our data indicate that the population size of *O. quelchii* is remarkably large (ca. 12 million individuals), with strong seasonal demographic fluctuations. Ecology and observed limited spatial movements challenge the likelihood of active dispersal among tepui tops in this species. Our results are counter to those predicted by the available genetic data but support two hypotheses of the OCBIL theory: reduced dispersibility and enhanced resilience. However, they do not support the expectation of a small refugial population size.

**Conclusion:**

We postulate that the insular, hostile tepui-summit environment tends to produce robust demographic populations, likely to buffer stochastic adverse environmental effects, rather than diversity, as observed in much younger post-Pleistocene Neotropical landscapes. Our results draw attention to the value of faunal studies using an OCBIL framework to better understand the ecology and evolution of this unique biota worldwide.

**Supplementary Information:**

The online version contains supplementary material available at 10.1186/s12862-024-02314-2.

## Background

It is generally accepted that population fitness positively correlates with genetic diversity [1], which in turn is determined by neutral processes (such as genetic drift; [2]) that are affected by population size, connectivity, and their interactions. Estimating the size and dispersal abilities of geographically isolated populations is of intellectual interest to evolutionary biologists but is also crucial for conservation [3].

**Fig. 1:**
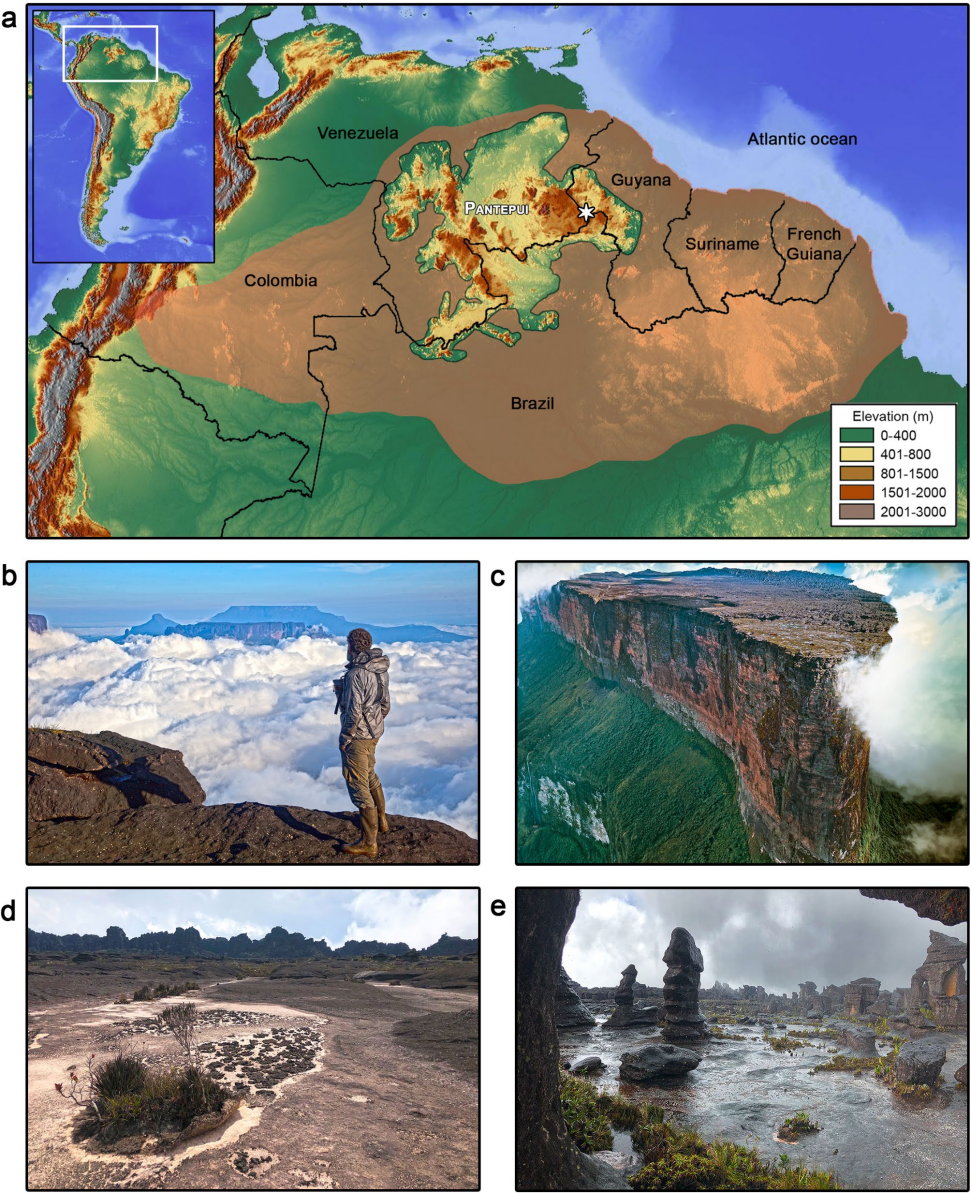
(**a**) Map of Pantepui and its location in South America (upper left inset). Orange shading refers to the Guiana Shield region; the star depicts the study site. (**b**) Western edge of the “Prow” on the summit of Roraima-tepui, showing the summit’s physiographical and ecological isolation from the surrounding uplands and neighbouring tepuis. (**c**) Drone photograph of the “Prow” of Roraima-tepui (taken on 14 August 2018, facing south). (**d**) The summit of Roraima-tepui in the dry season (photograph taken on 27 March 2019); note strong aridification. (**e**) The summit of Roraima-tepui in the wet season (photograph taken on 8 August 2018); note substantial flooding. Photos by PJRK

Tepuis are iconic Precambrian sandstone tabletop mountains scattered across the Pantepui biogeographical region [4]. Pantepui lies in the western Guiana Shield (Fig. 1a), and its old and nutrient-poor landscapes are one of the “OCBILs” (Old, Climatically Buffered, Infertile Landscapes) characterized by Hopper [5]. Hopper [5] was the first to develop the OCBIL theory, mainly to explain plant ecology in ancient landscapes and to better understand why these old landscapes challenge patterns of latitudinal gradients with regard to species diversity and endemism [6]. The author derived seven predictions from his theory (see [5] for details), and notably hypothesized that the biotic assemblages of OCBILs might display more complex population dynamics due to reduced dispersibility; the expected persistence of old lineages; refugial phenomena; inbreeding; adaptations to resource-limited, highly competitive environments; and high levels of resilience to lower evolutionary potential [5]. Later (e.g [6, 7]), more hypotheses (up to 12 in [7]) were derived from the OCBIL theory, with the proposition of ad-hoc mechanistic explanations and examples to challenge the hypotheses through direct and indirect evidence [7]. Testing the predictions of the OCBIL theory using different approaches and in different biogeographical regions is still in its early stages [7], especially in vertebrates for which studies using an OCBIL framework are scarce [8].

Tepui summits (Fig. 1b, c) constitute a unique landscape, reaching up to ca. 3 km elevation and being both physiographically and ecologically isolated from the more fertile surrounding environment and are believed to have served as the inspiration for Sir Arthur Conan Doyle’s *The Lost World*. Their characteristic vegetation grows on acidic, oligotrophic soils, and tepui summits are renowned for high floral and faunal endemism [9]. High tepui summits (> 2,500 m elevation) are further characterized by extreme and seasonally contrasting environmental conditions, typified by strong cold winds, intense solar, infrared, and ultraviolet radiation, with extreme droughts during the dry season contrasting with substantial precipitation and flooding during the wet season, which usually peaks over 2–3 months (Fig. 1d, e). Vertebrate life on high tepuis is usually depauperate, typically consisting of endemic early-branching lineages and a few ecologically plastic invaders (e.g [10–16], for amphibians and reptiles).

Toads in the genus *Oreophrynella* represent one of these endemic early-branching lineages seemingly well adapted to the tepui-top environment [17–20]. Terrestrial breeding and direct development have been reported in summit populations of *Oreophrynella*, with eggs sometimes deposited in large communal nests [20], though the general ecology of *Oreophrynella* species is largely unknown [18].

Although tepui-top *Oreophrynella* populations are predicted to have low connectivity and high genetic differentiation due to the ancient and complex geomorphology of the region, genetic divergence across isolated tepui-summit *Oreophrynella* populations is substantially lower than expected, close to or equal to zero in the two rapidly evolving mitochondrial gene fragments studied [21]. These contradictory features (low divergence vs. ancient isolation) contrast sharply with the many reports of strong structuring among vertebrate populations in post-Pleistocene Neotropical landscapes, which are not only much younger but are also much less topographically isolating (e.g [22–24], for amphibians). *Oreophrynella quelchii*, listed as Endangered by IUCN [25], reaches 16.2–29.8 mm snout-vent length [SVL] in adults [18] and is known only from the summit of two neighbouring tepuis, Roraima-tepui (∼ 2,800 m/summit area 35 km^2^) and Wei-Assipu-tepui (∼ 2,260 m/summit area 3 km^2^). These two summits are separated by less than 2 km (airline) and by dense highland and upland rainforests (see [26]). The uncorrected pairwise distance between these populations is 0% in 16S and ND1 gene fragments [21]. Good dispersal abilities are thus anticipated if gene flow is ongoing across these tepui summits and/or if vertical displacements were instigated by “recent” environmental stressors, such as climatic oscillations, as proposed by Rull [27]. However, the OCBIL theory posits that tepui summit populations, seemingly adapted to impoverished, patchily distributed soils, should have reduced dispersibility due to the substantial risk of individuals moving to sites unsuitable for establishment [7]. The OCBIL theory also predicts enhanced abilities to persist in small, fragmented populations (i.e., enhanced resilience; [5]). Indeed, millions of years of evolution in fragmented populations presumably selected for persistence and resilience to habitat fragmentation, possibly leading to inbreeding through small population size [5, 7].

These assumptions have yet to be empirically tested. To date, virtually nothing is known about the spatial ecology of tepui-top species or the drivers shaping space use patterns on tepui summits. Similarly, the population sizes and dispersal potential of tepui-top species have never been investigated. Therefore, the main aims of this work were to investigate, under the framework of the OCBIL theory, the population size and dispersal abilities of *Oreophrynella quelchii* using harmonic radar tracking and capture-mark-recapture (CMR) studies, which were also used to explore the species temporal niche and microhabitat use. Our field effort encompassed both wet and dry seasons, allowing us to assess whether seasonal variability and acute environmental variables/stressors can inform us about potential dispersal, population dynamics, and extinction risks of *O. quelchii* on the summit of Roraima-tepui. In a broader context, our results provide a framework for better understanding the global ecology and evolution of OCBIL faunas.

## Methods

### Study area

**Fig. 2:**
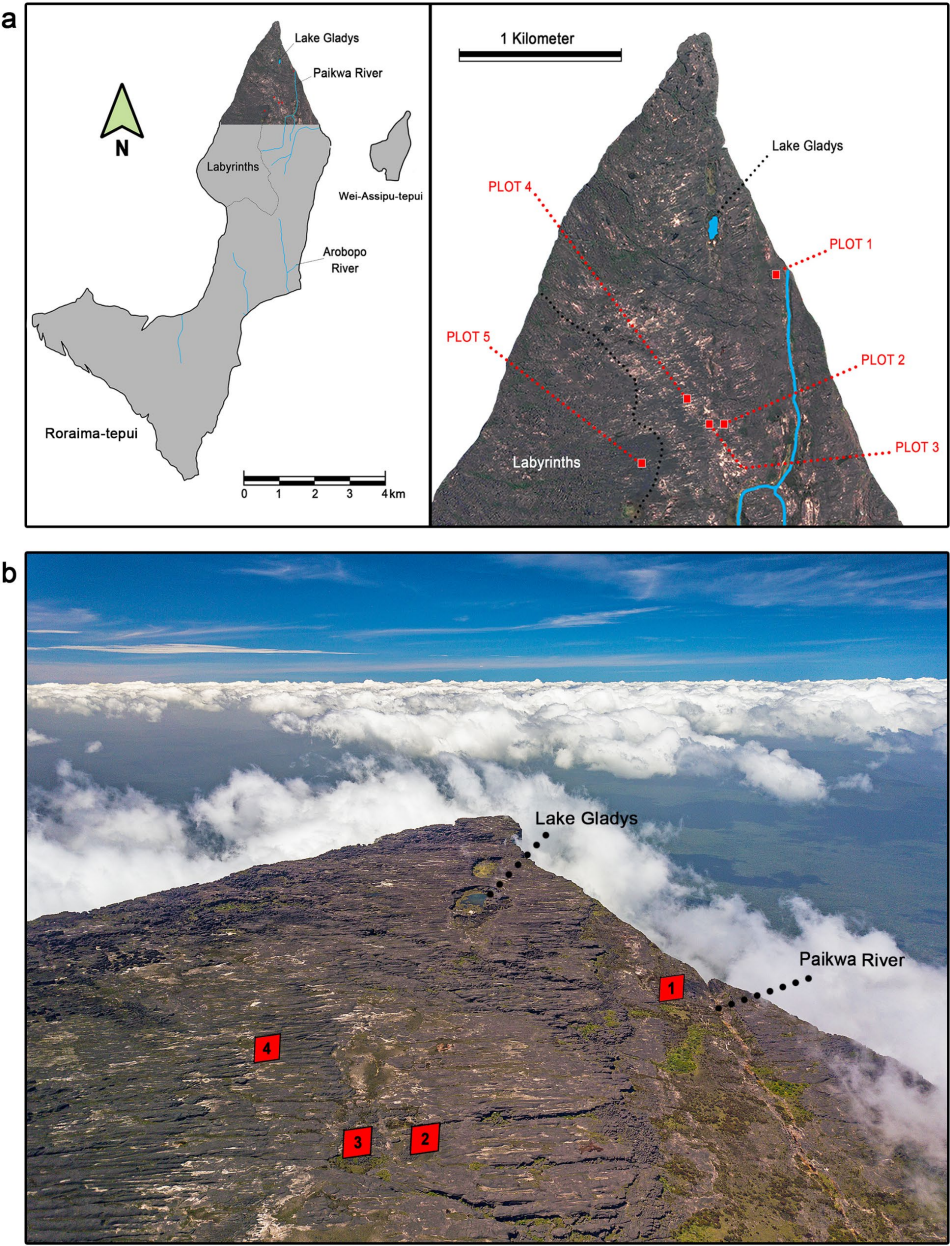
(**a**) Schematic of the summit of Roraima-tepui and Wei-Assipu tepui (left), and LANDSAT image of the “Prow” depicting the five 40 m × 40 m plots in red (right). (**b**) Drone photograph of the study area, showing four of the five plots (image shows an area of ∼ 2 km^2^). Photo by PJRK

Our study was conducted on the summit of Roraima-tepui (05°12’ N, 60°44’ W, Fig. 2a) in June–August 2018 (42 consecutive days/wet season) and February–March 2019 (40 consecutive days/dry season). The study area focused on the northern part of the tepui called “the Prow” (Figs. 1c and 2a and b). Summit vegetation includes low-growing tepui forests, tepui scrub, and high mountain meadows and grasslands [28]. The available literature reports the climate as submicrothermic and ombrophilous, with heavy rainfall, dense cloud and mist formation almost all year, and an average annual air temperature of 8–12 °C [29]. Minimum air temperatures of 1–2 °C have been recorded, and freezing temperatures may occasionally occur [29]. McDiarmid & Donnelly [30] reported mean annual precipitation of 2,500–3,000 mm at sites above 1,500 m elevation.

### Climatological monitoring

Precipitation was recorded every 24 h using a TFA^®^ Dostmann Rainman electronic rain gauge. Other climatic variables, including heat index, barometric pressure, relative humidity, temperature, wind chill, and wind speed, were recorded every 20 min using a KESTREL^®^ 4500 Weather Meter. Both devices were placed at the location of our field laboratory on exposed rock for most of the duration of each field campaign.

### Population parameters

**Fig. 3:**
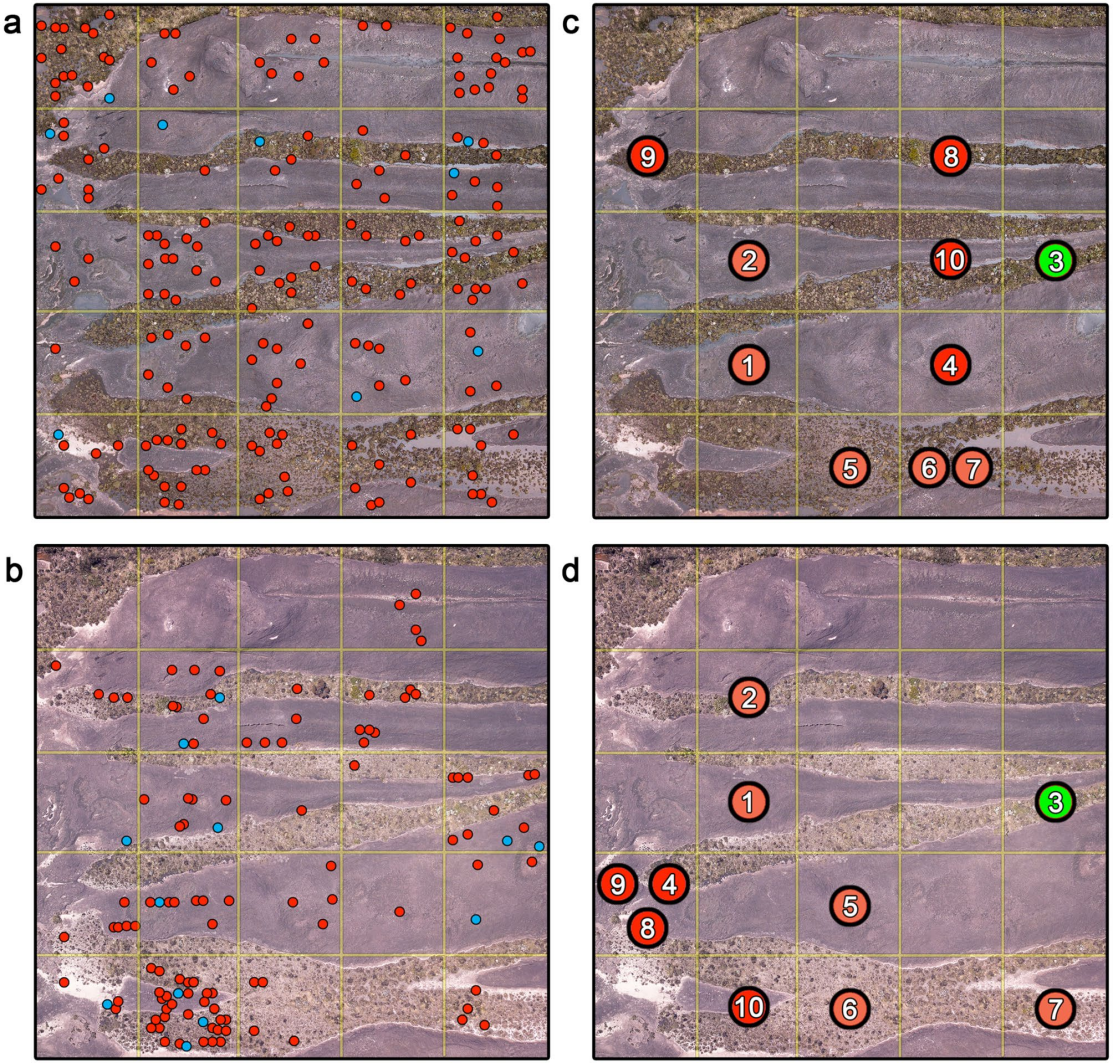
(**a**) Captures (red) and recaptures (blue) of *Oreophrynella quelchii* within Plot 4 during the wet season. (**b**) Captures (red) and recaptures (blue) of *O. quelchii* within Plot 4 during the dry season. (**c**,** d**) Recaptures of *O. quelchii* in Plot 4 across seasons. Each numbered circle represents a single individual captured (or recaptured) during both seasons (**c** = wet season; **d** = dry season). The green circle indicates a specimen found in the same quadrat, orange circles indicate specimens that moved to a neighbouring quadrat, and red circles indicate individuals that moved to a non-neighbouring quadrat. Note that (**a**, **b**) should not be compared to (**c**, **d**) as they represent different time frames

Population size was investigated using a CMR design modelled on Funk et al. [31] and Lettink [32]. A georeferenced LANDSAT-based map of the Prow was divided into numbered plots of 40 m × 40 m in QGIS 2.18 [33]; five plots were randomly selected using a random number generator (http://www.numbergenerator.org). Once in the field, the plots were delineated with a rope and further subdivided into 25 quadrats of 8 m × 8 m (Fig. 3); we thus surveyed a total of 125 8 m × 8 m quadrats. Each plot was mapped from the air (40–60 m altitude) using a DJI^®^ Mavic Pro drone (e.g., Fig. 3). Coordinates of the northeast corner of each plot are as follows: Plot 1: N5°14’16.3” W60°43’51.9”, 2649 m elevation; Plot 2: N5°14’01.5” W60°44’02.3”, 2680 m elevation; Plot 3: N5°14’00.7” W60°44’04.7”, 2680 m elevation; Plot 4: N5°14’06.7” W60°44’10.5”, 2680 m elevation; Plot 5: N5° 13’ 52.9” W60° 44’ 21.7”, 2654 m elevation.

Each plot was surveyed every five days with minimum disturbance (no substantial habitat modification or removal of vegetation) for five survey periods (totalling 25 days each in the wet and dry seasons). Each individual toad found within the plots was identified with a ventral surface photograph, taken in a glass petri dish, and assigned a unique identification number before being released at the exact location of capture (Fig. S1a). Notes on microhabitat variables and toad location within quadrats were recorded. Locations were later reported on the aerial maps taken by the drone to permit comparison of the distribution of individuals among plots between seasons. Drone footage was collected during wet and dry seasons to characterize differences in the landscape and hydric conditions.

### Population estimation

The ventral colour patterns of *Oreophrynella quelchii* are sufficiently variable to discriminate among individuals, allowing for unique identification through pattern recognition (Fig. S1a, b). CMR data were compiled from a manual photo-matching process (performed separately by three of the authors). Results of confirmed recaptures were processed to determine abundance using the Rcapture package (version 1.4-4) for capture-recapture experiments in R version 4.3.1 [34]. We used a closed population model for our analysis, which assumes that no birth, death, immigration, or emigration occurred during the study (i.e., population size within plots remained constant). This assumption is usually valid for data sampled across a relatively short period and when only adults are assessed [35, 36]. It should be noted that the data retrieved from both the tracking experiment (distances moved by individuals from initial sites of capture, non-linear movement patterns) and the plot surveys (distances moved by recaptured individuals within plots, absence of recaptures between close plots, occurrence of recaptures between seasons) favour the assumption of a closed population model for our data set (see further discussion in Appendix S2). To increase the robustness of our estimates, we pooled all data from the five plots in a single closed population for our analysis. Since our plot areas were highly representative of the habitat across the summit, we assumed that an extrapolation across the entire summit was reasonable [37]. Analysis of the closed population used the closedp.t function, which fits 12 combination models incorporating three sources of variation in capture probability (t = time-varying effect on p; b = behavioural effect on p; and h = heterogeneity in p for unknown reasons; with *p* = the probability of capture) that may have affected capture probability (see [34, 38, 39]; see also Table 1). Multinomial profile confidence intervals for the abundance were constructed using the function closedpCI.t. Since our single closed population was sampled during the rainy and dry seasons, population size for each season was estimated. After the mean density was determined for the closed population, the estimate was extrapolated to the full extent of the summit.

**Table 1:** Abundance estimates of the sampled closed population of *Oreophrynella quelchii* across seasons on Roraima-Tepui, using the closedp.t function in the rcapture package, which fits 12 combination models incorporating sources of variation that may have affected capture probability (t, b, h). Favoured models are in **bold-face** (see text for details). Warning #1 = model fit is questionable (algorithm did not converge, non-positive sigma estimate for a normal heterogeneous model or large asymptotic bias; Baillargeon & Rivest, 2007)

Wet season								
Model	abundance	stderr	deviance	df	AIC	ΔAIC	BIC	infoFit
**Mt**	**2981.2**	**615.4**	**13.334**	**25**	**80.679**	**0**	**104.368**	**OK**
Mth Chao (LB)	3108.4	689.1	13.008	24	82.353	1.674	109.989	OK
Mth Gamma3.5	7223.5	11233.7	13.034	24	82.379	1.7	110.016	warning #1
Mth Darroch	5248.7	5333.7	13.047	24	82.392	1.713	110.029	OK
Mth Poisson2	3755.6	1725.7	13.101	24	82.446	1.767	110.083	warning #1
M0	3027	625.7	41.632	29	100.977	20.298	108.873	OK
Mb	19750.5	180434.9	41.211	28	102.556	21.877	114.4	OK
Mh Chao (LB)	3153.5	700.3	41.323	28	102.669	21.99	114.513	OK
Mh Gamma3.5	7127.5	11068.3	41.349	28	102.695	22.016	114.539	warning #1
Mh Darroch	5232.1	5313	41.362	28	102.707	22.028	114.551	OK
Mh Poisson2	3785	1748.4	41.414	28	102.759	22.08	114.603	warning #1
Mbh	14,861	143067.1	41.209	27	104.555	23.876	120.347	OK

Dry season								
Model	abundance	stderr	deviance	df	AIC	ΔAIC	BIC	infoFit
Mbh	263.7	24.2	19.968	27	86.796	0	100.203	OK
**Mt**	**695.2**	**107.9**	**18.868**	**25**	**89.696**	**2.9**	**109.807**	**OK**
Mth Chao (LB)	733.9	131.7	18.422	24	91.25	4.454	114.713	OK
Mth Gamma3.5	1127.4	973.9	18.522	24	91.349	4.553	114.812	warning #1
Mth Darroch	936	529.3	18.56	24	91.388	4.592	114.851	OK
Mth Poisson2	776.6	224.6	18.675	24	91.503	4.707	114.966	warning #1
Mb	336.2	51.7	28.878	28	93.706	6.91	103.761	OK
M0	705.6	109.9	35.829	29	98.656	11.86	105.36	OK
Mh Chao (LB)	743.6	134	35.421	28	100.249	13.453	110.304	OK
Mh Gamma3.5	1115.6	960.7	35.517	28	100.344	13.548	110.4	warning #1
Mh Darroch	934.9	528.5	35.553	28	100.381	13.585	110.436	OK
Mh Poisson2	783	227.9	35.66	28	100.488	13.692	110.543	warning #1

### Breeding patterns

A total of 135 specimens were collected during both seasons (117 specimens in the wet season; 18 specimens in the dry season) for a separate study. Individuals were euthanised by immersion in 10 ml of 2% Linisol^®^ (lidocaïne hydrochloride; an amide class anaesthetic) for a few minutes and dissected for gonad analysis, and the number of ovarian eggs was determined.

### Harmonic radar tracking

Across the two field seasons, a total of 100 adult individuals (55 females and 45 males) were collected by hand at different locations on the Prow (within a ca. 1 km radius from the field laboratory, but never within sampling plots) and were equipped with harmonic tracking reflectors [40–42]. Coordinates of each initial collection location were recorded with a GARMIN^®^ 60CSx GPS unit and marked with a flag. Subsequent coordinate positions were marked by moving the flag and making location calculations using Pythagoras’ theorem based on the distance and angle of locations. This method was an alternative to location estimation based on GPS coordinates due to the potential error of hand-held GPS devices and the relative inaccuracy of GPS over distances shorter than 3 m [43].

Two types of harmonic reflectors were used for tracking: (1) a manufactured RS-30-CL RECCO^®^ transponder (hereafter called RECCO), and (2) a hand-made transponder (hereafter called DIODE) (Fig. S1c; descriptions can be found in Appendix S1). Post mounting, the reflectors weighed 0.05–0.07 g.

Captured individuals were measured (SVL) with digital callipers and weighed using a precision digital scale to the nearest 0.01 g before and after tagging. Specimens were kept in small plastic boxes for 8–24 h post-tagging on wet absorbent paper to monitor health before release at their initial collection site. Once released, specimens were located approximately every 24 h (once a day, but see below) using a RECCO^®^ Detector 98B.

During the wet season, 22 females and 17 males were tracked approximately once every 24 h using RECCO reflectors. Individuals weighed 0.38–2.48 g, and the weight% of the reflectors ranged between 2.8% and 18.4% (16 individuals exceeded the 10% recommended proportion). From these 39 individuals, 243 points of contact were recorded throughout the study. Sixteen individuals were omitted from further analysis because tracking lasted less than five days, leaving 23 individuals tracked for up to 19 consecutive days, with 203 points of contact for analysis.

During the dry season, 25 females and 25 males were captured and fitted with reflectors for tracking approximately once every 24 h. Individuals weighed 0.47–1.96 g with reflector weight% between 3.6% and 13.2% (seven above the 10% recommendation). The first 30 individuals were fitted with DIODE reflectors before making a decision to use RECCO reflectors for the remaining 20 individuals. This change occurred due to frequent entanglement and subsequent death of individuals associated with the longer DIODE reflectors. From these 50 individuals, 205 points of contact were made over the duration of the study. Sixteen individuals were tracked for five or more days (up to 12 consecutive days) and were thus included in the data analysis with 139 points of contact.

During the dry season only, an additional cohort of 11 individuals (eight females, three males) was tracked using RECCO reflectors approximately every 12 h, thus during both the day and night, to account for potential differences in diel activity. Individuals weighed 0.47–1.96 g with reflector weight percentages between 4.2% and 9.2% and were tracked for up to 10 consecutive days.

### Space use and movement modes

A Gaussian generalised linear model (GLM) was fitted to data for the mean daily distance moved by males and females in the wet and dry seasons, including a measure of body size (weight).

Analysis of the total distance moved by individuals during the daytime for both study periods was performed using generalised linear mixed models (GLMMs). Data for the total distance moved contained a high proportion of zero counts (16%), though with responses distributed equitably among treatment combinations. Consequently, a zero-altered (hurdle) model with Gamma distribution was employed [44]. Zero-altered models are partitioned into two parts, with a binary process modelling zeros and positive counts and a second process modelling only positive counts using a zero-truncated model [45]. This modelling approach enabled us to separately identify the environmental variables associated with movement in *O. quelchii* (binary part), and the distance traversed when an individual moved (zero-truncated part). Because multiple observations were obtained for each individual throughout the study, a random intercept for individual toads was included in models to introduce a correlation structure between observations for different records for the same individual. Analysis was performed on individuals tracked for five or more days, to ensure that harmonic reflector did not inhibit behaviour and movement. Due to an imbalance in the data, two levels of habitat type, on rock and under rock, were dropped from the analysis.

To overcome variance inflation in the model, we combined six abiotic environmental variables (wind speed, wind chill, air temperature, relative humidity, heat index, and barometric pressure) in one PCA to obtain the major axes of environmental variation. Variables were scaled and centred before analysis.

### Habitat selection

Habitat type was divided into four categories: (i) on rock, (ii) under rock, (iii) associated with vegetation, and (iv) in/on mud. The vegetation category (iii) was subcategorized based on plant functional groups with respect to the degree of protection they provided against UV/solar irradiance, drought and potential predators (see Table S1 for species listing). The three subcategories increased in complexity, incorporating elements from the previous category, and were as follows: (x) providing shelter, (y) protecting against drought, and (z) potentially enhancing anti-predator defense. A chi-square test was used to test the observed distributions and habitat associations of individuals.

All analyses were performed using the R Statistical Language Environment (version 4.3.1) [46].

## Results

### Climatological factors

Ground-based climate data are scarce in the Pantepui highlands, and our data highlight the previously undocumented magnitude of seasonal variation on high tepui summits. Precipitation during the wet season was more regular and occurred at much higher rates than precipitation during the dry season (Fig. S2 shows 24-hour averages). We recorded a total of 1,124.4 mm of rainfall over 35 days in the wet season (10/07–13/08/2018) and a total of 171.8 mm of rainfall over 35 days period in the dry season (26/02–1/04/2019; i.e., 85% lower precipitation compared to the wet season). Maximum precipitation in 24 h was 95.3 mm on 29/07/2018 (wet season). Daily average temperature, barometric pressure, and relative humidity fluctuated minimally during the wet season. While more variation was evident during the dry season, the means of temperature and barometric pressure remained similar across both seasons. The minimum air temperature was 6.5 °C on 26/02/2019 at 0500 (dry season); the maximum was 20.4 °C on 27/03/2019 at 1220 (dry season). Air temperature during the wet season ranged from 8.7 °C to 16.3 °C. Wind chill showed greater fluctuation during the wet season than the dry season, and relative humidity showed greater fluctuation during the dry season. The lowest relative humidity recorded was 14% on 22/03/2019 at 1320 (dry season). Drone footage showed marked differences in hydric conditions within each surveyed plot between seasons, with a moist environment during the wet season (large areas of plots filled with water) and a xeric environment during the dry season (almost no water visible; Fig. S3).

### Breeding pattern

Dissection of sacrificed individuals showed ovarian eggs in 90% of mature females (45/50) collected in the wet season and 100% (10/10) of mature females collected in the dry season. Likewise, egg clutches (deposited under rocks, under moss, or deep in vegetation), with eggs at different developmental stages, as well as juveniles, were found in both seasons and during separate field campaigns, notably in July 2015 and November 2019. These findings imply that breeding is continuous. The number of vitellogenic eggs ranged between 4 and 13 (mean = 7.5) per individual.

### Temporal niches

Diel activity rhythms were tested during a 14-day period in the dry season only. Based on our data set, toads were active both day and night, though activity was 8% higher at night. Notably, wind speed, air temperature, and barometric pressure were lower at night.

### Habitat selection

During both seasons, we found *Oreophrynella quelchii* primarily associated with vegetation, mainly with 23 plant species (Table S1). Chi-square goodness of fit tests among four habitat types show that *O. quelchii* was associated with vegetation, irrespective of season (wet season, χ2 = 259.01, *P* < 0.001; dry season, χ2 = 322.9, *P* < 0.001) (Table S2, Fig. S4), associating most strongly with the greatest vegetation complexity (wet season, χ2 = 77.43, *P* < 0.0005; dry season, χ2 = 72.92, *P* < 0.001) (Table S2, Fig. S4).

### Population parameters

During the wet season, 384 individuals were captured across all five surveyed plots, with 21 recaptures (e.g., Fig. 3a for Plot 3). During the dry season, 212 individuals were captured with 31 recaptures (e.g., Fig. 3b for Plot 3). Although juveniles were omitted from CMR data due to the lack of unique ventral surface patterning (the ventral surface is black in juvenile *Oreophrynella quelchii*), 69 juveniles were captured during the wet season, and 27 during the dry season. No individual was found in a plot outside the one in which it was initially identified, even when plots were close to each other (e.g., Plots 2 and 3 separated by less than 80 m, Fig. 2). Recapture rate of individuals between seasons was 6%; 64% of these were found in either the same or a neighbouring quadrat (e.g., Fig. 3c, d for Plot 3). One male specimen, first captured on 17/7/2018 (wet season), was recaptured on 1/03 and 21/03/2019 (dry season) up to 248 days later and in the same 8 m × 8 m quadrat.

### Population size

Analysis of capture history used the best-fitting (lowest AIC value) model from Rcapture [31]; however, predictive models contain uncertainty and results from capture-recapture experiments should always be interpreted cautiously. Model Mt gave the best fit for the wet season dataset, while model Mbh scored slightly higher than Mt for the dry season dataset (ΔAIC = 2.9; Table 1), suggesting that individuals behaved differently in relation to capture across seasons, an assumption that seems unlikely. Indeed, while a behavioural response to initial capture (e.g., boldness-shyness) may be observed in mammals, especially while using traps [36], it is less realistic for an amphibian. Moreover, the estimate of 263.7 individuals in the closed population (model Mbh) seems too low and would imply a ca. 11-fold decrease in population size during the dry season, which is less realistic than the ca. 4-fold decrease using the Mt model (see below). Since the Mt model scored second best (and first for the wet season data set), and to help select the most biologically meaningful estimate, we used the function *uifit* from Rcapture, which is used to discriminate between close estimators by producing fit statistics for each model for the number of new captures on each occasion and if the experiment was continued (see [34] for details). Estimates using the *uifit* function indicate that both Mt and Mbh models are close to the observed *ui* (i.e., the numbers of first captures at each capture occasion; Table 2) but that the Mt predicted values for *ui* are almost constantly closer to the observed *ui* than those of the model Mbh, leading us to favour the Mt model over the Mbh model. After averaging, the population size of *Oreophrynella quelchii* across the entire summit of Roraima-tepui was estimated to be 12,811,707 (95% CI: 8.80 × 10^6^–19.84 × 10^6^) and 2,987,622 (95% CI: 2.25 × 10^6^–4.14 × 10^6^) in the wet and dry seasons, respectively. This is a density of 0.4 (wet season) and 0.09 (dry season) individuals per square meter, with a 4.3-fold decrease in population size in the dry season compared to the wet season.

**Table 2:** Estimates obtained using the *uifit* function in Rcapture to discriminate between close estimators (see Baillargeon & Rivest, 2007 and main text for details). The predicted values for *Ui* closest to the observed *ui* are in bold-face

	observed	Mt	Mbh
*u1*	47	47	47
*u2*	59	**58.74058**	64.5186
*u3*	55	**51.72137**	45.31117
*u4*	29	**30.93959**	31.82186
*u5*	21	22.59847	**22.34837**

### Space use

**Fig. 4:**
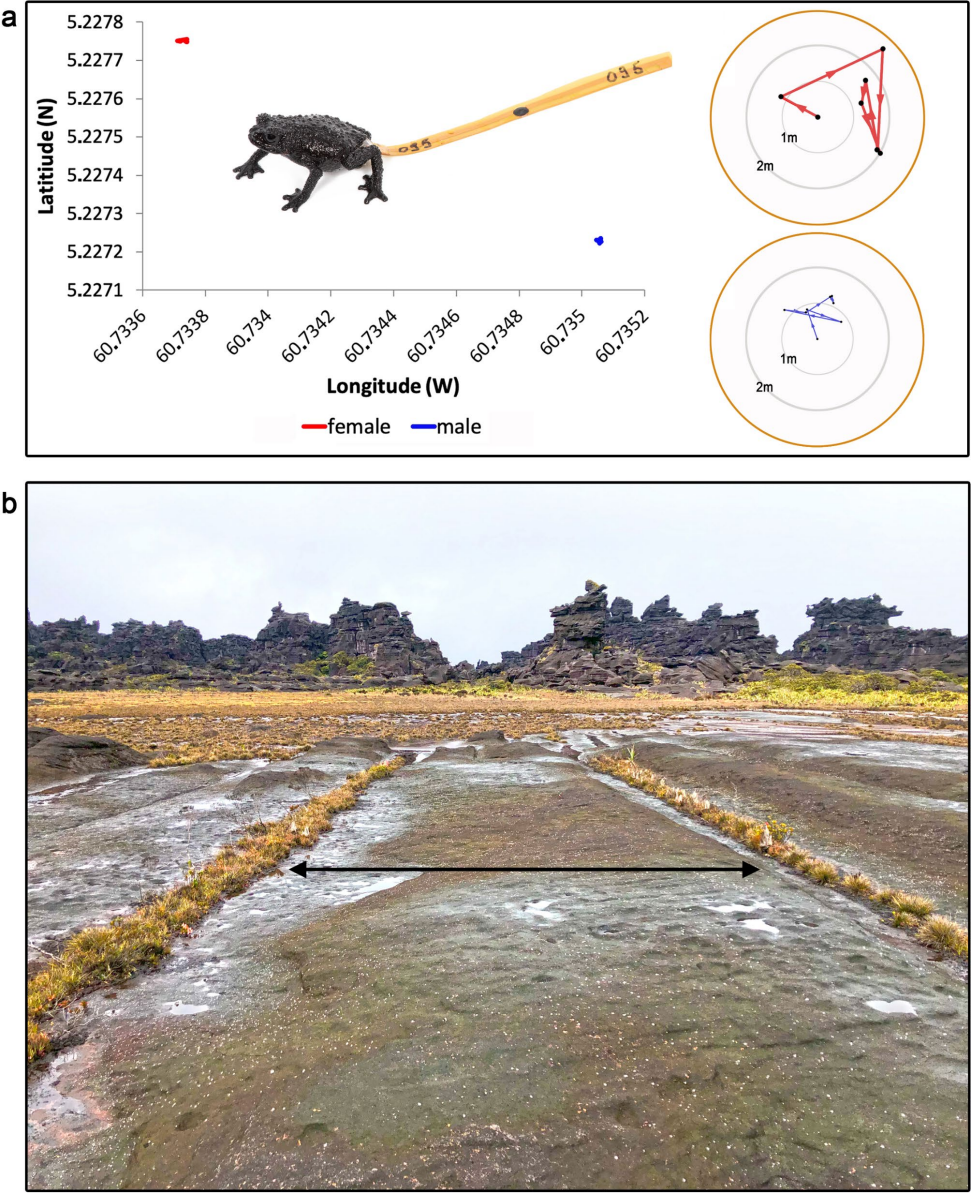
(**a**) Directional movement of two *Oreophrynella quelchii* (male and female) during tracking in the dry season. The circles on the right side are zoomed-in insets of two randomly selected coloured spots (one blue, one red). Circles show point-by-point movement and trajectories from the start location (normalised to the centre of the circle) of each individual. Numbers written on tags (in this case 095) served to identify tracked individuals without having to capture them. (**b**) Example of typical back-and-forth movement between vegetation patches. Photos by PJRK

There was no significant relationship between the relative weight of the harmonic tracking reflectors as a function of toad body weight and average distance moved (*P* = 0.342), and the slope of the relationship did not vary significantly between seasons (*P* = 0.214). Likewise, there was no significant relationship between the type of harmonic tracking reflector (DIODE vs. RECCO) on the average distance moved (*P* = 0.381). See Appendix S2 for the fate of tracked individuals and further discussion about the potential impact of reflectors. For individuals in both seasons and all tracking periods, data showed a non-linear movement pattern of *Oreophrynella quelchii* (Fig. 4a). The maximum distance travelled in 24 h was 30 m, point-to-point, by a male individual during the wet season. The greatest distances traversed were often within 24 h following release, suggesting an effect of disturbance. However, removal of distances moved in the first 24 h made no qualitative difference to the main outcomes of the study and the complete data set was retained for analysis. After an initial extended movement, individuals tended to move much shorter distances, though some regularly moved 6–10 m between the same vegetation patches, sometimes crossing open rocky areas (Fig. 4b).

### Relationship between sex and movements

**Fig. 5:**
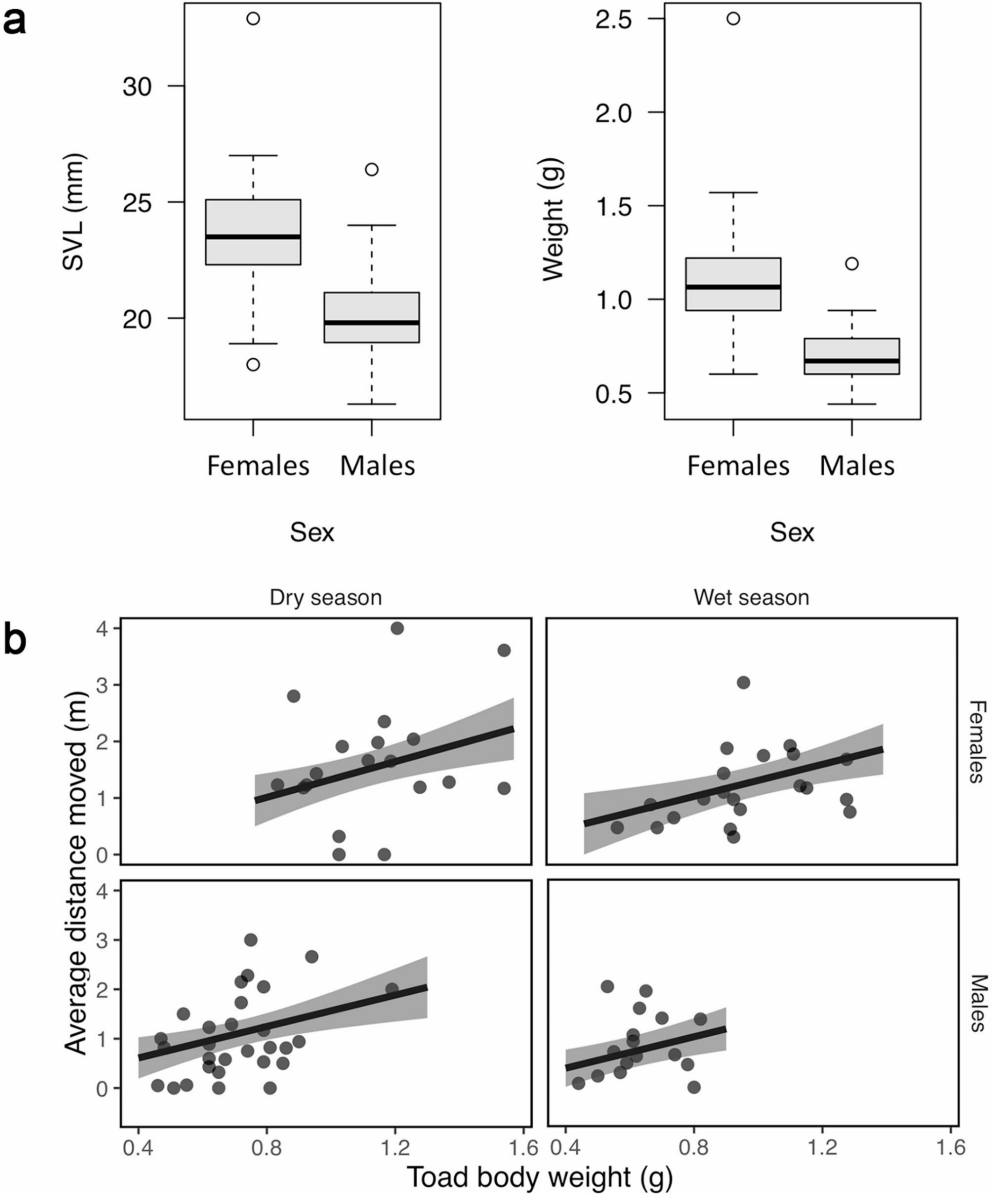
(**a**) Comparative SVL (left) and weight (right) between sexes. (**b**) Mean distance moved by *Oreophrynella quelchii* as a function of body weight. Shaded areas are 95% confidence intervals. Dark points are observed data for individual toads

Females were, on average, larger than males, both by SVL (*t*_87_ = 7.75, *P* < 0.001; Fig. 5a) and weight (unpaired t-test, *t*_87_ = 8.38, *P* < 0.001; Fig. 5a). Tracking data showed no significant sex x season interaction in mean daily distance moved, and this parameter was dropped from the analysis. There was no significant effect of sex or season on the mean daily distance moved, though there was a significant positive effect of body size (weight), irrespective of sex (Tables S3–S4, Fig. 5b).

### Relationship between environmental factors, activity, and movements

**Fig. 6:**
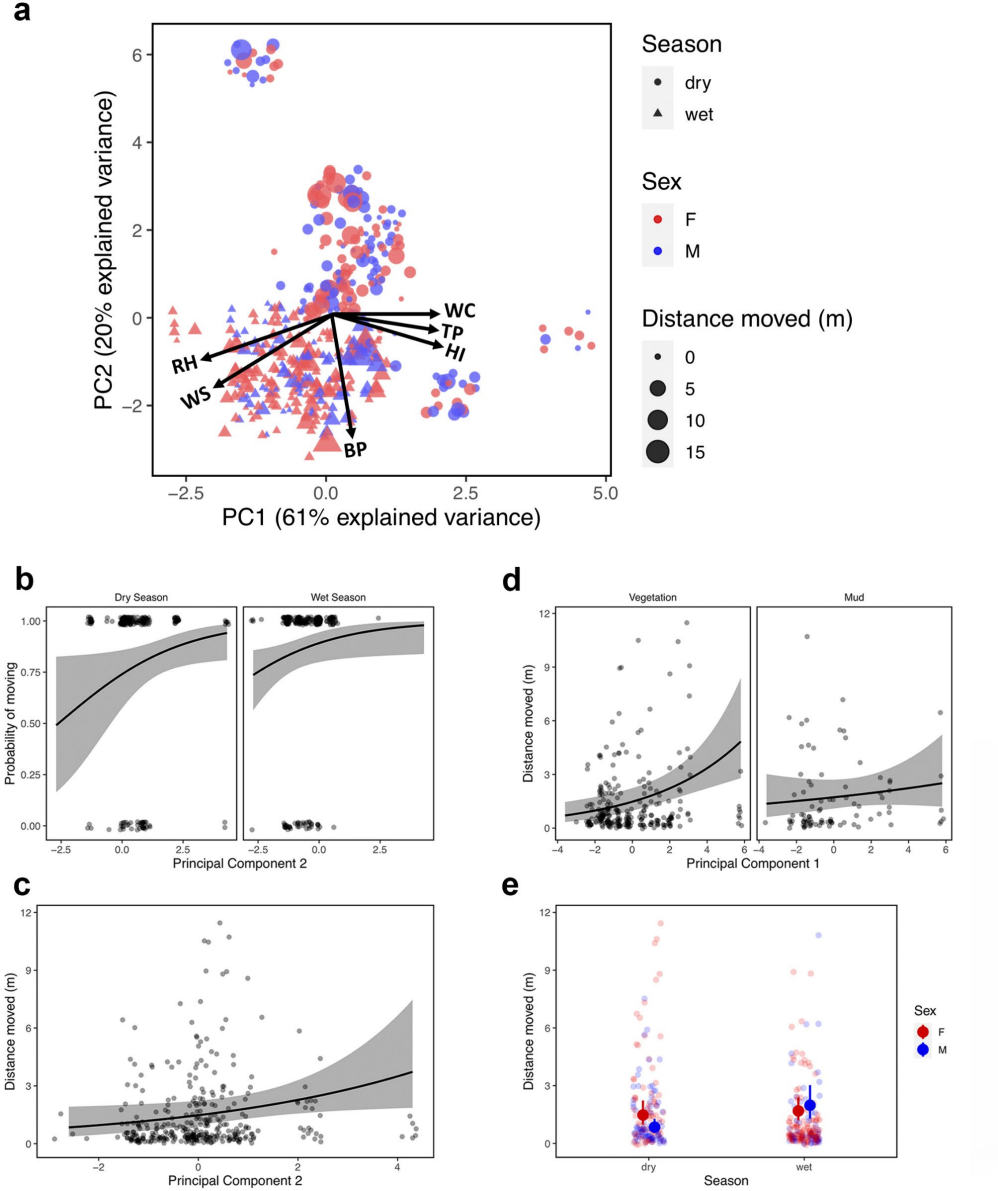
(**a**) PCA biplot and loadings for the plot of PC1 against PC2 for *Oreophrynella quelchii* (WS = wind speed, WC = wind chill, TP = air temperature, RH = relative humidity, HI = heat index, BP = barometric pressure). (**b**) Mean probability of movement in *Oreophrynella quelchii* as a function of PC2 in the dry and wet seasons. Shaded areas are 95% confidence intervals. Dark points are observed data for individual toads. (**c**) Mean distance moved by *O. quelchii* as a function of PC2. (**d**) Mean distance moved by *O. quelchii* as a function of PC1 on vegetation and mud habitats. (**e**) Mean distance moved by *O. quelchii* in the dry and wet seasons for males and females. Error bars are 95% confidence intervals. Coloured points are observed data for individual toads

The first PC of abiotic variables (“PC1”) explained 61.3% of the variance among individuals (Table S5 provides details on loadings and percentages explained by each of the PC axes), while PC2 explained 19.8% of the variance (Table S5). A plot of PC1 against PC2 indicated that these variables effectively discriminated among environmental factors between seasons in *Oreophrynella quelchii* (Fig. 6a).

The probability that a toad moved was positively associated with PC2 (*P* = 0.038) (Fig. 6b, Table S6), though not with other environmental variables (Table S6). In the case that *O. quelchii* did move, the distance they moved was positively associated with PC2 (*P* = 0.032) (Fig. 6c, Table S6) and with a habitat x PC1 interaction (*P* = 0.029); greater movement distances in response to PC1 were associated with vegetation than with mud (Fig. 6d, Table S6). Total individual distance moved was significantly associated with a sex x season interaction (*P* = 0.011) (Table S6), with females moving greater distances in the dry season and males moving greater distances in the wet season (Fig. 6e).

## Discussion

Few animal populations have been studied under the framework of the OCBIL theory, which was initially proposed for plants [5]. Studies on vertebrates using an OCBIL framework are limited [8], with only one study ever conducted in the Pantepui biogeographical region, focusing on diet specialization, one of the OCBIL predictions [18]. The present work represents the first analytic evaluation of the spatial ecology and population biology of a tepui summit vertebrate at multiple spatial scales.

The use of harmonic radar tracking (100 individuals tracked) and CMR data (596 individuals captured/52 recaptured) allowed us to provide insights about the temporal niche, microhabitat use, population size, and dispersal abilities of the tepui-summit endemic toad *Oreophrynella quelchii* across the wet and dry seasons on the summit of Roraima-tepui.

Our results additionally provide insights into the climatological characteristics of seasonal variation on a high tepui summit. Although it remains to be determined if the 2019 dry season was exceptionally pronounced for the region, the available literature seemingly underrates seasonal variation on high tepui summits. Our record of 1,124.4 mm of precipitation over only 35 days in the wet season suggests that the mean annual precipitation of 2,500–3,000 mm reported by McDiarmid & Donnelly [30] may be underestimated.

Observations during both seasons show increased daytime activity of *Oreophrynella quelchii* during the wet season and increased nighttime activity during the dry season. Cathemerality (i.e., the exploitation of both the diurnal and nocturnal niche; [47]) seems common among tepui summit anurans (Kok, pers. obs.) and suggests plasticity depending on the most suitable environmental conditions.

Although higher relative humidity usually stimulates movement and dispersal in amphibians (e.g [48]), only lower barometric pressure and, to a lesser extent, lower windspeed implied a higher probability of movement in *Oreophrynella quelchii*. Loadings for PC1 additionally indicated that higher temperature/windchill/heat index and lower relative humidity (relationship higher in vegetation than on mud) predicted greater movement over 24 h. Our results also showed a marginally greater probability of (non-linear) movement in the wet season, during which males moved more than females. Changes in the activity of *O. quelchii*, as influenced by barometric pressure, could relate to an individual’s state of hydration. This finding could explain why larger individuals (females, which are expected to have higher water storage due to their larger size and subsequent lower surface area to volume ratio) have a greater propensity to move further than small individuals (males, which are expected to have lower water storage capacity) in the dry season. Our preliminary observations on the thermal physiology of *O. quelchii* suggest high resilience to dehydration in this species, which is corroborated by the present data indicating an overall low impact of harsh climatological factors on *O. quelchii* displacement and activity. Resilience and avoidance are not mutually exclusive, and further research is warranted to elucidate the mechanisms involved in such associations. Likewise, evaporative water loss in amphibians is faster at high wind speeds [49], which could explain a higher probability of movement at lower wind speeds.

During both seasons, *Oreophrynella quelchii* was most often associated with vegetation. The vegetation type most preferred was complex vegetation that provided protection against drought and possibly enhanced predator defense. Individuals were found the least often associated with categories (i) and (ii), i.e., on rock and under rock, respectively. Interestingly, *O. quelchii* densities were lower but more stable across seasons in heavily vegetated areas than in rocky areas with sparse vegetation patches. This could be linked to hydric conditions that make heavily vegetated areas too damp for egg deposition after heavy rainfall but more amenable to survival during drought episodes.

Overall, our results are counterintuitive to those predicted by the available genetic data but support two assumptions of the OCBIL theory: reduced dispersibility and enhanced resilience. However, they reject the expectation of a small refugial population size. Indeed, our findings suggest a remarkably large population size of *O. quelchii*, though with strong demographic fluctuations across seasons (a fourfold decline was observed during the dry season). Our field observations during harmonic radar tracking in the dry season suggest an increase in mortality due to prolonged severe drought, with several individuals seemingly unable to relocate to a more favourable location (in moister microhabitats). Movement to “distant” hypothetical shelters implies hazardous crossing of inhospitable areas that can locally reach ground temperatures > 60 °C during the day (Fig. S5). Although *O. quelchii* individuals regularly cross dry ground at temperatures in the 30 °C range, “hot spots” in the 40–70 °C range could act as ephemeral but effective barriers to movement. Spatial aggregation in moist environments has been routinely observed in our plots in the dry season (Fig. 3b). Such seasonal variation in population size implies elevated recruitment (the addition of new individuals to a population). The year-round breeding capability of *O. quelchii* suggests a potential for continuous recruitment (see also Appendix S3). The higher number of subadults/juveniles found within plots during the wet season compared to the dry season suggests that recruitment may be higher during the wet season.

The observed sharp contrast in genetic diversity and biodiversity between tepui summits (ancient, topographically highly isolating pre-Pleistocene landscapes) and much younger, less topographically isolating post-Pleistocene Neotropical landscapes (the surrounding upland/lowland forests) is worth highlighting. Our results suggest that the insular, hostile tepui summit environment tends to produce robust demographic populations (i.e., high abundance, which is presumably necessary to buffer the adverse effects of isolation and harsh environment) rather than intrinsic biological diversity (high number of species). Post-Pleistocene Neotropical landscapes usually produce a high level of diversity rather than high population sizes (e.g [22–24, 50, 51], for amphibians). This assumption of increased population sizes to buffer the effect of insularity and extreme environment would benefit from further testing in other OCBIL faunas.

Low levels of genetic diversity among *Oreophrynella* summit populations might reflect gene flow, possibly implying either contemporary active dispersal across tepui tops, passive dispersal, or recent historical dispersal instigated by environmental stressors such as Pleistocene climatic oscillations (i.e., disturbance-vicariance; [52, 53]). Testing these hypotheses requires a broader genetic sampling within and among tepui summits and high-throughput population genomics (in progress). Nevertheless, in addition to ecological requirements, limited dispersal behavior and high adult philopatry, as demonstrated by our tracking and CMR results, challenge the hypothesis of ongoing active dispersal across tepui summits in *O. quelchii*.

Although adverse and highly contrasting between seasons, climatological factors have a seemingly low impact on toad displacement and activity. This finding suggests high resilience and adaptation of *O. quelchii* to the hostile tepui-top environment and further questions the potential of dispersal instigated by environmental stressors. Some pre-Miocene lineages (such as *Oreophrynella*) possibly became too highly adapted to tepui summit environments to survive long enough outside their ecological niche and actively disperse across unsuitable habitats. Direct developers have no obligate dependency on standing water, freeing them from seasonal migration, which also likely influences their spatial ecology.

Although active long dispersal of adults is not supported by our data, active dispersal of juveniles cannot be ruled out, even if improbable. Long-distance dispersal along steep inclines with large elevation gains has been reported in juveniles of the frog *Rana luteiventris* [54]. However, juveniles of that species are the size of (or larger than) an adult *O. quelchii* and, as opposed to *Oreophrynella*, disperse in continuous habitat. Owing to their tiny size and weight (< 5 mm, < 0.01 g; [14]), juvenile *Oreophrynella* might prove to be propagules well adapted for wind-mediated dispersal, both within a single summit and across geographically close tepui tops.

Mountaintop habitats and species cannot shift further and are at risk of extirpation due to global climate change [3, 55]. Although *Oreophrynella quelchii* is seemingly resilient to the harsh tepui-summit environment, their long-term survival is questionable in the face of an increase in temperature and/or the duration and intensity of drought episodes on the summit of Roraima-tepui. If repeated, climate-change-induced anomalies could severely deplete *O. quelchii* population size and place them at risk of extinction.

## Conclusions

Our results show that the population size of *Oreophrynella quelchii* is remarkably large with strong seasonal demographic fluctuations, and that active dispersal among tepui summits is not a credible explanation for the observed low genetic divergence among tepui summit populations. Our work supports two assumptions of the OCBIL theory, reduced dispersibility and enhanced resilience but rejects the expectation of small refugial population size.

We postulate that the insular, hostile tepui summit environment tends to produce robust demographic populations, likely to buffer stochastic adverse environmental effects, rather than diversity as observed in much younger, much less topographically isolating, post-Pleistocene Neotropical landscapes.

The processes we describe are likely to exist across other ancient, hostile, insular landscapes and other faunal populations, and our results draw attention to the potential value of faunal studies using an OCBIL framework for a better understanding of the ecology and evolution of this unique biota.

## Electronic supplementary material

Below is the link to the electronic supplementary material.

Supplementary Material 1

## Data Availability

Data supporting the analyses in this contribution, including R scripts, are either in the Supporting Information or are available on figshare at 10.6084/m9.figshare.25341532. Supplemental videos are available on figshare at 10.6084/m9.figshare.26490712.
