# Supplementary material for: Lost in space and time: robust demography and enhanced resilience buffer adverse environmental effects in a highly isolated and sedentary pre-pleistocene landscape vertebrate
Source: BMC Ecol Evol. 2024 Oct 11;24:125. doi: 10.1186/s12862-024-02314-2 (PMC11468910; doi:10.1186/s12862-024-02314-2)
Supplement: Supplementary file 1 — Supplementary Material 1 [file 12862_2024_2314_MOESM1_ESM.pdf]

Supporting Information for

Lost in space and time: robust demography and enhanced resilience buffer adverse environmental effects in a highly isolated and sedentary pre-Pleistocene landscape vertebrate

This PDF file includes:

Appendices S1 to S3
Tables S1 to S6
Figures S1 to S6
Videos S1 to S2 (https://doi.org/10.6084/m9.figshare.26490712)
Supplementary References

Appendix S1. Description of harmonic reflectors

The RECCO reflector contained a copper antenna on a Polyimide (PI) carrier with a cover layer of PI material; the reflectors were 66 mm (length) × 3 mm (width) × 0.05 mm (height). The DIODE reflector consisted of a Schottky diode (surface mount "S2") bonded with conductive epoxy (CHEMOTRONICS® CircuitWorks) to two antennas; the antennas were 30 mm (anode) and 130 mm (cathode) long and made of 0.15 mm ultra-flexible steel fishing leader. The diode and connecting points were sealed using non-toxic aquarium silicone. Both reflector types were glued to silicone tubing (diameter 0.4 mm) using non-toxic aquarium silicone, and the tubing was fixed around the waist of *Oreophrynella quelchii* individuals with cotton thread. Depending on the landscape and vegetation cover, the range of detection for all reflectors was 7–17 m. On wet surfaces, the range dropped to ca. 4 m, and dropped further if the reflector was submerged (0.7 m in 10 mm depth of water and no detection in 0.2 m).

Appendix S2. Fate of tracked individuals and potential impact of harmonic reflectors

<u>Wet season</u>

Of the 39 tracked individuals, 34 were found dead or dying during the experiment: 11 probably died from firefly larvae predation (see details in Kok et al. 2019), 11 from complications surrounding the reflector tag (e.g., entangled in vegetation), and 12 due to less identifiable causes (see below). Two individuals lost the reflector tag, and their fate is unknown.

<u>Dry season</u>

Of the 50 tagged individuals, 46 died during the experiment: two were evidently predated by firefly larvae (see details in Kok et al., 2019), 11 died from complications associated with the reflector tag (mostly entanglement in vegetation), and 33 died from other causes; some were found drowned in deep pools or desiccated due to a severe drought during the tracking period. The remaining four individuals lost their reflector, and their fate is unknown.

<u>Dry season night/day (cohort of 11 individuals)</u>

One reflector was lost during tracking and four individuals were found dead (one due to predation by firefly larvae and three due to complications associated with the reflector).

One might wonder whether the mortality of tracked individuals should be considered as natural or related to the harmonic reflector. The impact of the harmonic reflectors on predation is unknown, but we assume that carrying the reflectors might have impaired some of the smallest individuals from escaping predators or made them more conspicuous in their natural environment. Exhaustion is a possible cause of death for the smallest specimens with weight percentage of reflectors ranging above the 10% recommended proportion; these specimens were usually tracked for less than 5 days and therefore not included in our analyses (see main text). Necropsies indicated that a possible explanation for the death of specimens tracked over longer periods (> 7 days) is the cotton thread used to secure the reflector around the waist mechanically obstructing proper defecation, although no tissue swelling could be observed in the field. This condition did not seemingly impact movement of individuals, nor their behaviour. Indeed, (1) there was no correlation between weight percentage/harmonic reflector type and average distance moved (see main text); (2) tracked individuals behaved in a similar way to observed untracked individuals. We even observed a small male (ca. 18 mm SVL) equipped with a harmonic reflector guarding a large egg clutch (Fig. S6).
See also Video S1 and Video S2, (available on figshare at 10.6084/m9.figshare.26490712) taken after 7 and 4 tracking days, respectively.

It must be noted that previous studies reporting the tracking of very small anuran species (< 30 mm SVL) are scarce (e.g., Altobelli et al., 2023; Borzée et al., 2016, 2019; Garrido-Priego et al., 2024; Gourret et al., 2011; Kim et al., 2019; Pašukonis et al., 2014, Pašukonis, Loretto & Hödl, 2014, Pašukonis et al., 2018; Roznik & Alford, 2015). Many remain unclear about the exact number of tracking days and the fate (mortality) of tracked individuals, making comparisons difficult, especially regarding mortality rate of individuals tracked more than 5 days (in only 30% of the studies mentioned above, with only one mentioning a tracking period exceeding 7 days). We tracked individuals (ca. 18–25 mm SVL) for up to 19 consecutive days. If our tracking periods had been kept at 5 days maximum, the mortality rate would have been reduced by up to 50%. Therefore, we assume that the mortality rate observed during tracking is mostly related to the harmonic reflectors and should not violate our postulate of a closed population (i.e., that the population size within our plots remained constant over the 25 days of sampling).

Appendix S3. Reproductive output

The species breeds all year round, most collected females had developed eggs, and the number of eggs can reach up to 13 per female (mean 7.5, see main text). If every female only produced two clutches per year (our data clearly suggest more) and the survival rate of those clutches was 50%, a fourfold increase of population size between seasons is easily achievable. We hypothesize that the breeding strategy of *O. quelchii* (e.g., communal nests, clutch attendance, continuous breeding) correlates with high reproductive output allowing rapid population expansions, which is key in thriving on tepui summits.

Table S1. Vegetation associated with the presence of *Oreophrynella quelchii* on the summit of Roraima-tepui.

Subcategory	Description	Plant ID	Family
**x**	Refuge against UV irradiance [no obvious advantage against drought detected; *Oreophrynella* individuals mostly found exposed under the plant, rarely in/on the plant]	*Stomatochaeta condensata*<br>*Bonnetia roraimae*<br>*Cyrilla racemiflora*<br>*Ledothamnus* sp<br>*Maguireothamnus speciosus*<br>*Clusia* sp	Asteraceae<br>Bonnetiaceae<br>Cyrillaceae<br>Ericaceae<br>Rubiaceae<br>Clusiaceae
**y**	**x** + refuge against drought [*Oreophrynella* individuals mostly found hidden at base of plant/under roots]	*Xyris decussata*<br>*Nietneria corymbosa*<br>*Stegolepis guianensis*<br>*Cladium costatum*<br>*Carex* sp<br>*Connellia augustae*<br>*Tillandsia turneri*<br>*Drosera roraimae*<br>*Lycopodium* sp<br>*Cortaderia roraimensis*<br>*Epidendrum* sp<br>*Octomeria* sp<br>Moss/sphagnum<br>Lichen	Xyridaceae<br>Nartheciaceae<br>Rapateaceae<br>Cyperaceae<br>Cyperaceae<br>Bromeliaceae<br>Bromeliaceae<br>Droseraceae<br>Lycopodiaceae<br>Poaceae<br>Orchidaceae<br>Orchidaceae<br>Bryophita<br>unknown
**z**	**x** + **y** + enhancing anti-predator defence ["spiny plants"; *Oreophrynella* individuals mostly found hidden inside the plant or patch of plants]	*Orectanthe sceptrum*<br>*Connellia caricifolia*<br>*Connellia quelchii*	Xyridaceae<br>Bromeliaceae<br>Bromeliaceae

Table S2. Chi-square test among four habitat types (top) and three vegetation categories based on complexity (bottom). Letters indicate statistically significant differences among groups (note: habitat and vegetation complexity were analysed separately).

	**Season**	
**Habitat**	**Wet**	**Dry**
i	6[c]	3[f]
ii	11[c]	1[f]
iii	162[b]	159[e]
iv	64[a]	42[d]

**Vegetation Complexity**	**Wet**	**Dry**
x	2[b]	4[d]
y	79[a]	89[c]
z	85[a]	65[c]

Table S3. Results of Gaussian GLM to test the effects of sex, season, and body weight on the mean daily movement of *Oreophrynella quelchii*.

	**Gaussian GLM**		
**Coefficient**	**Estimates**	**CI (95%)**	**P**
Intercept	-0.32	-1.51 – 0.87	0.593
Season (wet)	-0.22	-0.60 – 0.15	0.237
Sex (male)	0.29	-0.25 – 0.84	0.282
Weight	1.59	0.58 – 2.60	**0.002**
Observations	85		
R^2 / R^2 adjusted	0.187 / 0.157		

Table S4. Comparative body size and movement measurements between the sexes across both seasons. Statistically significant p-values are emboldened.

Variable	**Wet Season**							**Dry Season**						
	Male			Female				Male			Female			
	Mean ± SE	Range		Mean ± SE	Range		*P*	Mean ± SE	Range		Mean ± SE	Range		*P*
Body mass (g)	0.63±0.03	0.44	0.82	1.060.08	0.60	2.50	**<0.001**	0.74±0.03	0.46	1.19	1.17±0.04	0.87	1.57	**<0.001**
SVL (mm)	18.95±0.39	16.30	21.50	23.3±0.67	18.00	32.9	**<0.001**	20.59±0.36	17.8	26.4	24.15±0.34	21.5	26.90	**<0.001**
Mean distance (m)	1.44±0.43	0.10	7.04	1.22±0.19	0.19	3.70	0.7475	1.18±0.21	0	5.50	1.18±0.31	0	5.86	**0.0066**
Maximum distance (m)	5.28±1.76	0.50	30.00	4.49±0.83	0.30	14.50	0.866	2.57±0.49	0	12.50	5.07±1.02	0	18.30	**0.02932**
Displacement (m)	4.99±1.54	0.44	25.42	4.52 ±0.86	0.18	13.61	0.7446	2.63±0.55	0	13.95	5.30±1.13	0	17.02	**0.0279**
Total distance (m)	8.53±3.13	0.50	56.35	8.67±1.31	0.15	21.22	0.2127	3.86±0.76	0	20.45	10.02±2.45	0	46.99	**0.0189**
Number of movements	6.25±0.44	1.00	9.00	8.57±0.94	1.00	21.00	0.1183	3.43±0.55	0	10.00	4.85±0.87	0	13.00	0.2305

Table S5. Loadings of environmental variables and the proportion of variance explained by each PC axis.

	**PC1**	**PC2**	**PC3**	**PC4**	**PC5**	**PC6**
Wind speed	-0.358	-0.421	0.238	0.751	-0.268	-0.050
Wind chill	0.519	0.001	0.081	-0.085	-0.830	-0.167
Air temperature	0.499	-0.176	0.220	0.179	0.158	0.784
Relative hygrometry	-0.365	-0.223	0.687	-0.555	-0.133	0.143
Heat index	0.468	-0.259	0.423	0.064	0.443	-0.578
Barometric pressure	0.029	-0.822	-0.488	-0.292	-0.001	0.001
sd	1.917	1.091	0.795	0.707	0.044	0.013
Variance explained	0.613	0.198	0.105	0.083	0.001	0.001
Cumulative variance explained	0.613	0.811	0.916	0.999	0.999	1.000

Table S6. Results of zero-altered gamma GLMM to test the effects of sex, habitat, season, precipitation, PC1 and PC2 on total distance moved by individual *Oreophrynella quelchii*.

	**Bernoulli model**			**Gamma model**		
**Coefficient**	**Odds Ratios**	**CI (95%)**	**P**	**Estimates**	**CI (95%)**	**P**
Intercept	2.85	1.35 – 6.02	**0.006**	1.48	0.98 – 2.23	0.064
$Sex_{(male)}$	0.65	0.38 – 1.12	0.119	0.57	0.39 – 0.84	**0.004**
$Habitat_{(mud)}$	1.40	0.76 – 2.57	0.275	1.17	0.89 – 1.53	0.262
$Season_{(wet)}$	2.89	0.83 – 10.04	0.095	1.14	0.59 – 2.20	0.689
Precipitation	0.88	0.61 – 1.26	0.478	0.91	0.75 – 1.10	0.328
PC1	1.15	0.92 – 1.42	0.215	1.23	1.10 – 1.37	**<0.001**
PC2	1.49	1.02 – 2.18	**0.038**	1.24	1.02 – 1.51	**0.032**
$Sex_{(male)}$ x $Season_{(wet)}$				2.05	1.18 – 3.58	**0.011**
$Habitat_{(mud)}$ x PC1				0.87	0.77 – 0.99	**0.029**
**Random Effects**						
σ^2	3.29			1.63		
τ	0.13_{ind}			0.12_{ind}		
ICC	0.04			0.07		
N	80_{ind}			80_{ind}		
Observations	427			345		
Marginal R^2 / Conditional R^2	0.041 / 0.077			0.066 / 0.132		

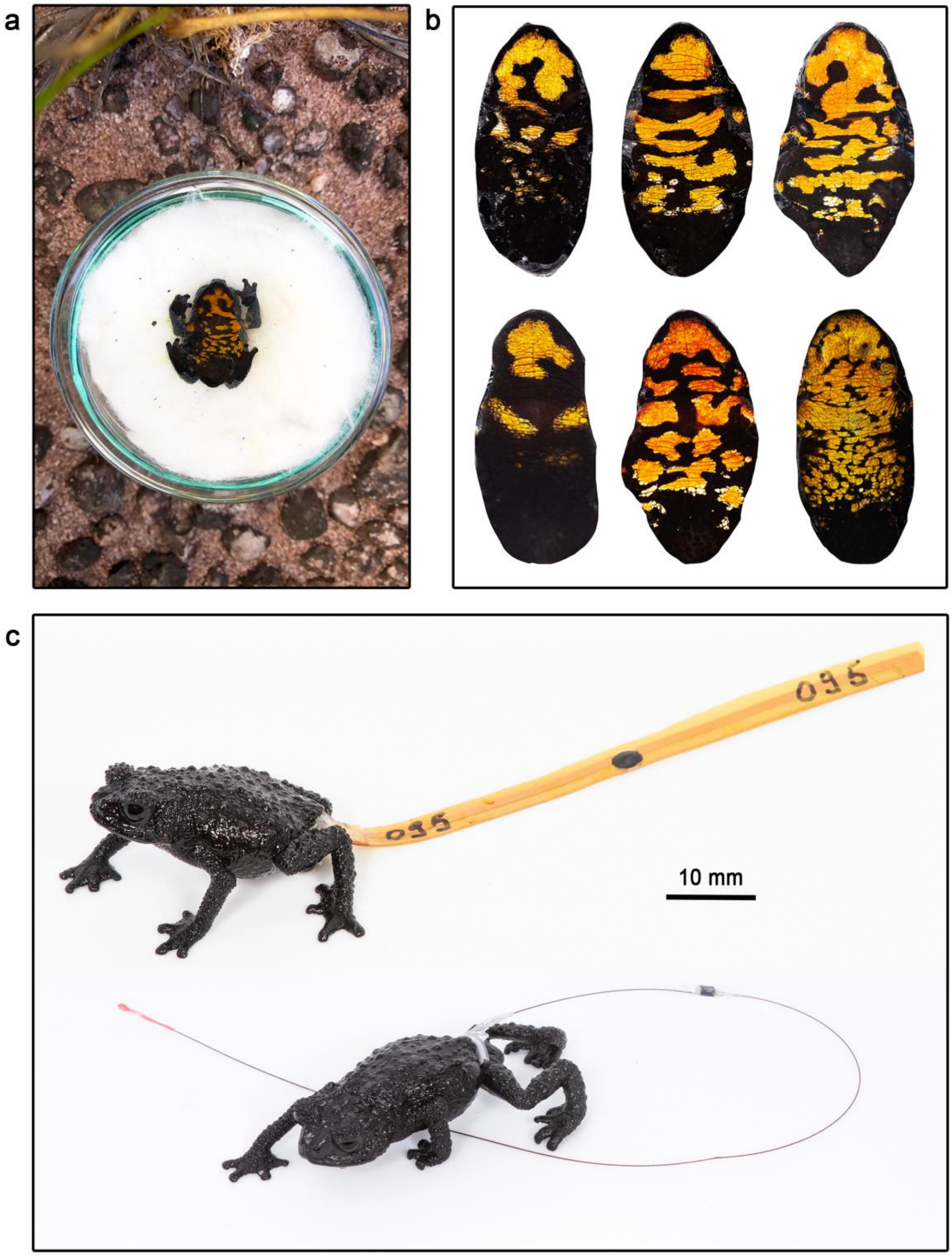

Figure S1. (**a**) An individual of *Oreophrynella quelchii* in a 60 mm Petri dish used for ventral pictures. (**b**) Variation in ventral coloration and patterning of *O. quelchii*. (**c**) Individuals fitted with reflector tags: RECCO (top) and DIODE (bottom).

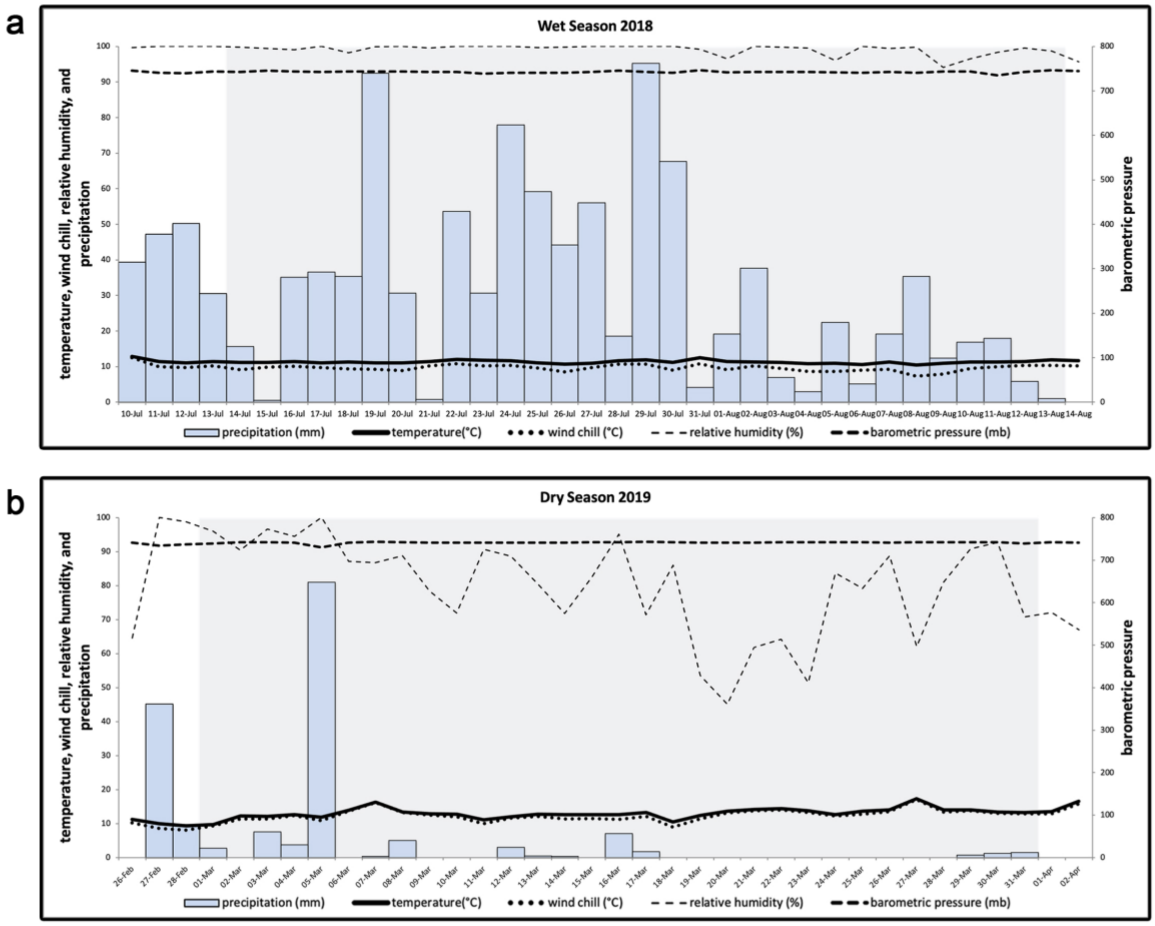

Figure S2. Climatic variables (24-hour averages) during the wet (**a**) and dry (**b**) seasons. Grey highlighted areas indicate tracking days.

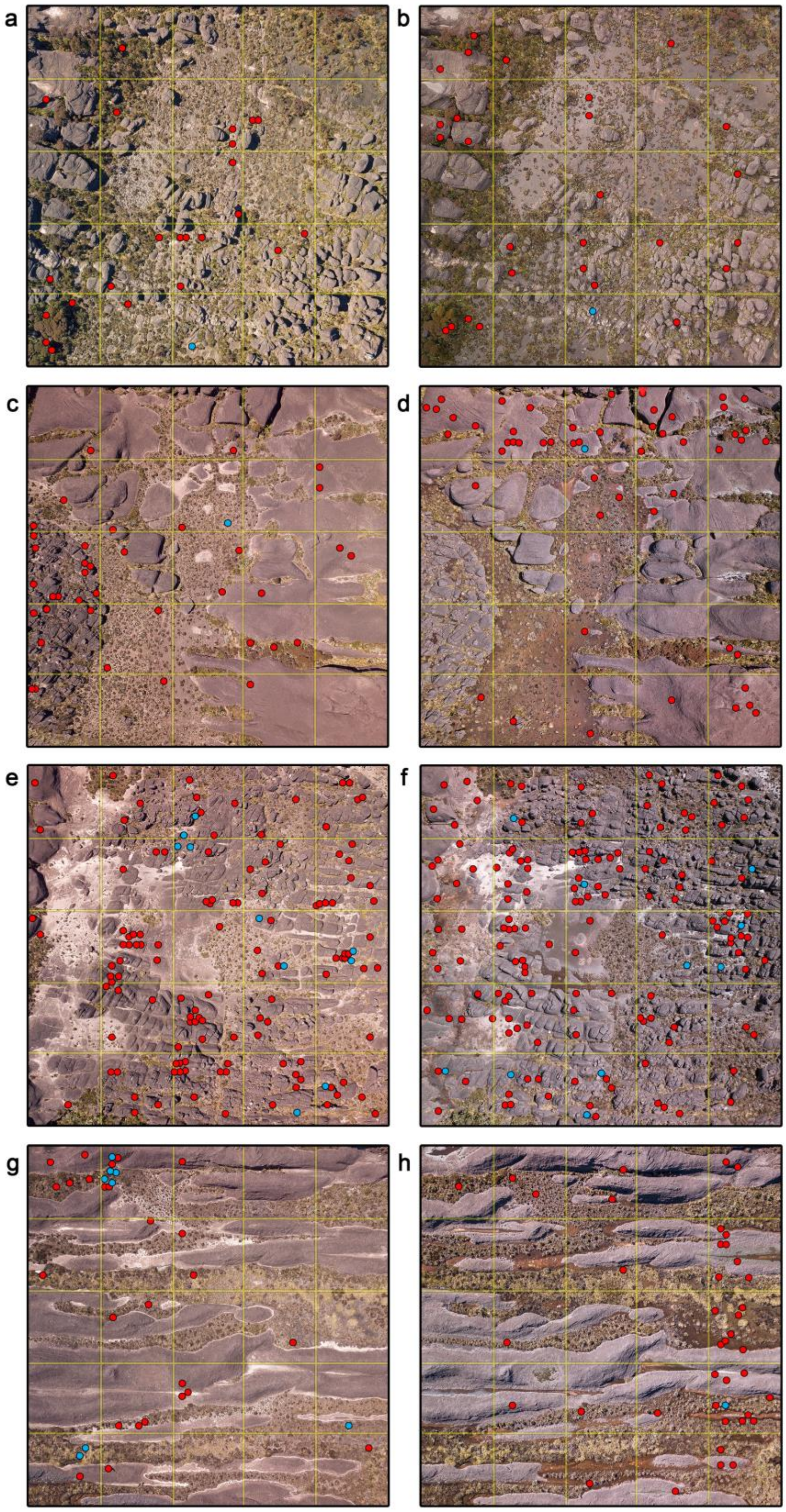

Figure S3. Captures (red) and recaptures (blue) of *Oreophrynella quelchii* within (**a**) Plot 1 during the dry season. (**b**) Plot 1 during the wet season. (**c**) Plot 2 during the dry season. (**d**) Plot 2 during the wet season. (**e**) Plot 3 during the dry season. (**f**) Plot 3 during the wet season. (**g**) Plot 5 during the dry season. (**h**) Plot 5 during the wet season.

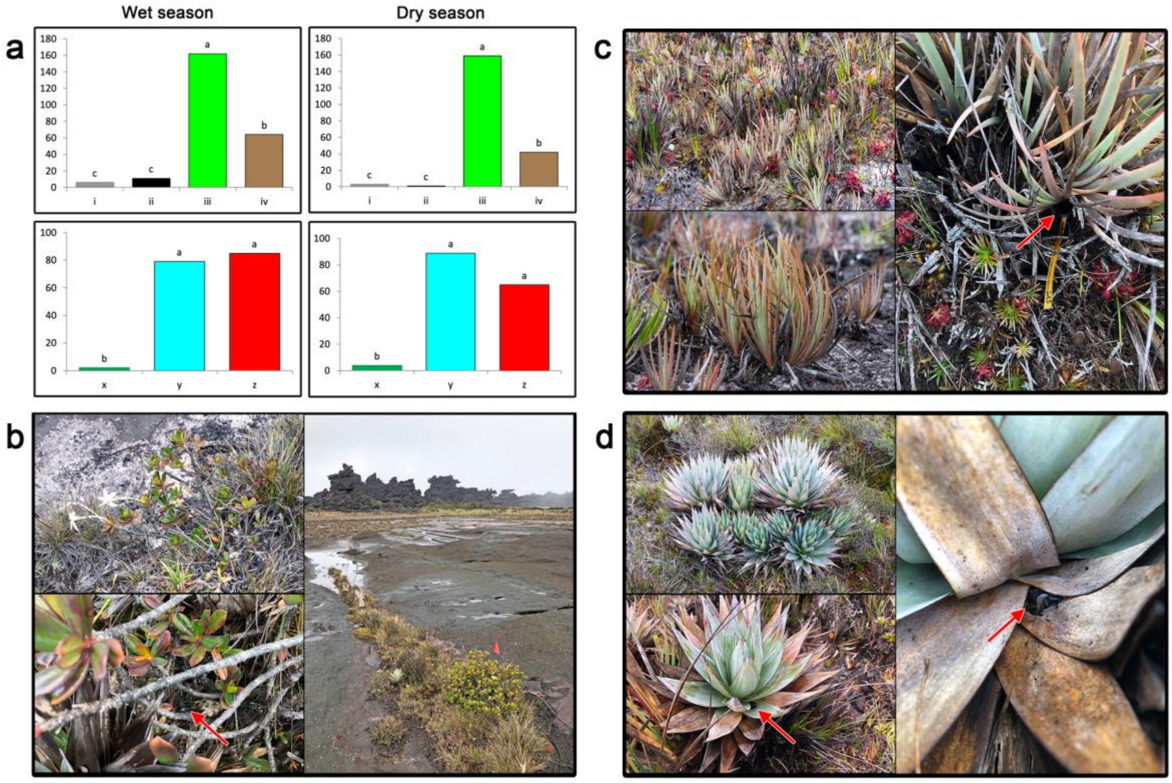

Figure S4. (**a**) Distribution of *Oreophrynella quelchii* among four habitat types (upper) and three subcategories (lower) in the wet and dry seasons on the summit of Roraima-tepui (see text and Table S3 for details). (**b**) Example of subcategory x: *Maguireothamnus speciosus*. (**c**) Example of subcategory y: *Nietneria corymbosa*. (**d**) Example of subcategory z: *Orectanthe sceptrum*. Red arrows indicate a tracked individual of *Oreophrynella quelchii*.

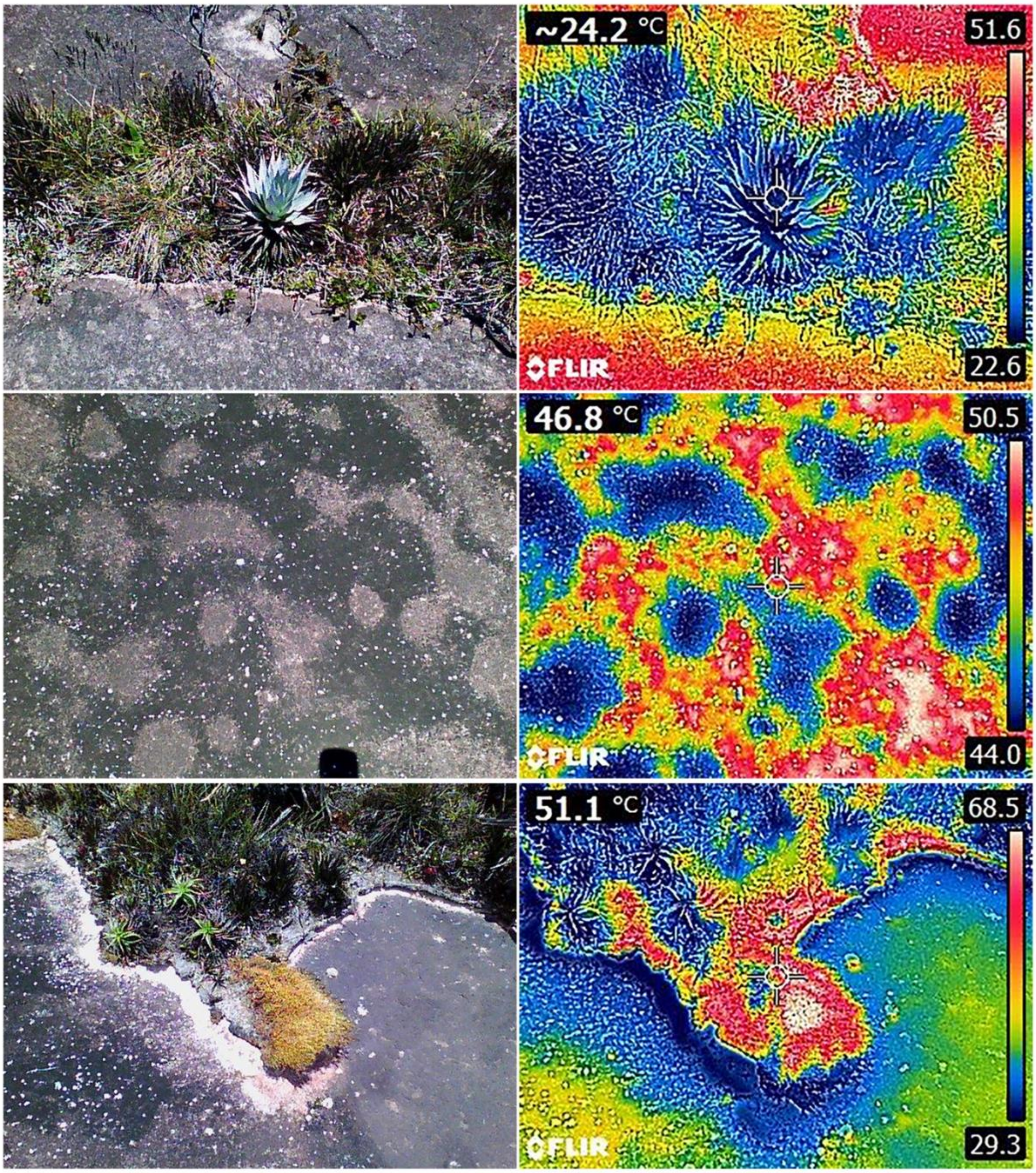

Figure S5. Photos (**left**) and corresponding thermal images (**right**) showing thermal profiles of tepui summit soil during the day in the dry season. Note "hot spots" where temperatures can reach almost 70°C. The upper right thermal image shows how individuals may be "trapped" in small cooler vegetation islands. All images taken with a FLIR E8 infrared camera at ca. 100 cm distance from the ground.

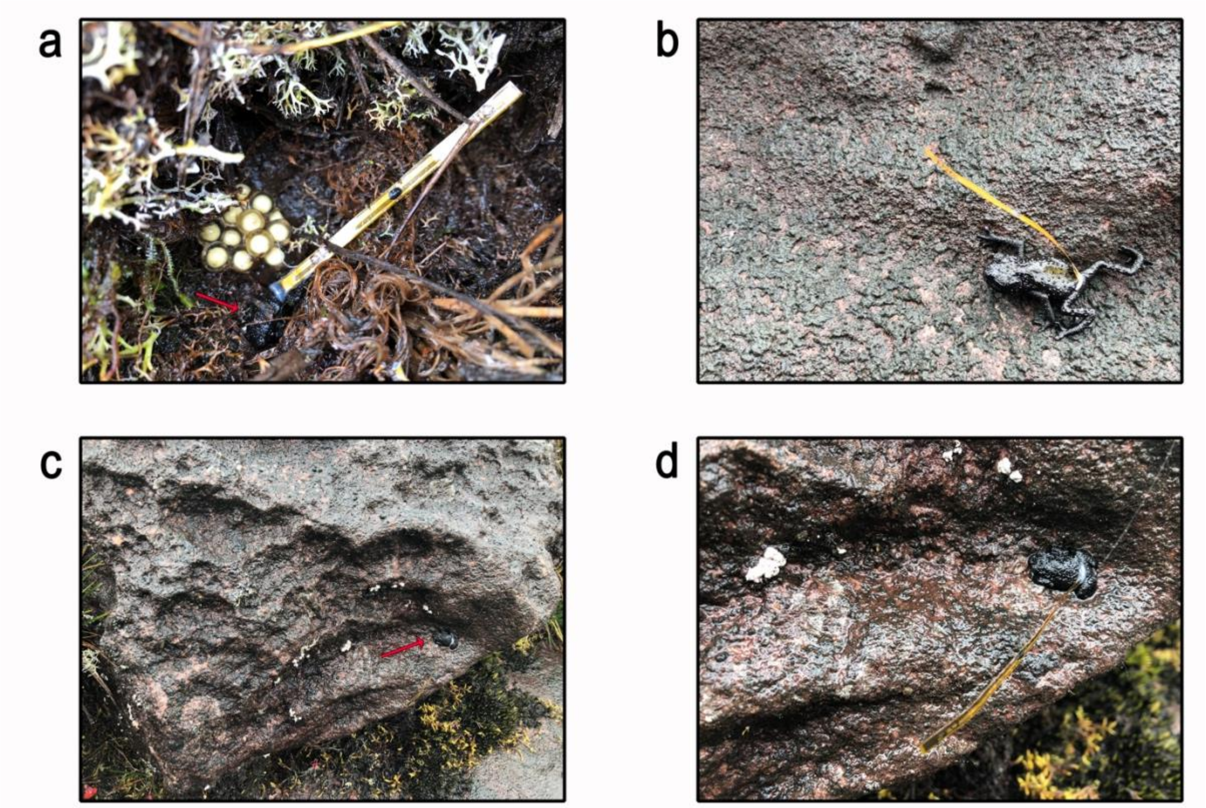

Figure S6. Observed behaviour of individuals equipped with harmonic reflectors. (**a**) One of our smallest tagged adult males *Oreophrynella quelchii* (ca. 18 mm SVL) found guarding a clutch of 13 eggs laid deep in the vegetation. The specimen (indicated by a red arrow) is seen trying to bury itself deeper in the vegetation. Another tagged individual (female) was found nearby (a small piece of its yellow harmonic reflector is seen at the bottom of the image). (**b**) A tagged female observed walking by day on a vertical rock wall (see also Video S1). (**c**) and (**d**) A tagged male (indicated by a red arrow in (**c**)) observed resting on a large rock during the day. These behaviours were regularly observed in untagged individuals.

Video S1. available on figshare at https://doi.org/10.6084/m9.figshare.26490712
A female *Oreophrynella quelchii* filmed while moving on open rock during the wet season, after 7 days of tracking. Unfortunately, the individual was injured by the harmonic reflector (the urostyle pierced the skin) on the 10th day of tracking and had to be euthanized.

Video S2. available on figshare at https://doi.org/10.6084/m9.figshare.26490712
A female *Oreophrynella quelchii* filmed while moving on open rock during the wet season, after 4 days of tracking. This individual managed to remove the harmonic reflector on the 5th day of tracking.

Supplementary References

Altobelli, J. T., Bishop, P. J., Dickinson, K. J. M., & Godfrey, S. S. (2023). Suitability of radio telemetry for monitoring two New Zealand frogs (*Leiopelma archeyi* and *L. hamiltoni*). *New Zealand Journal of Ecology*, 47(2): 3532.

Borzée, A., Choi, Y., Kim, Y. E., Jablonski, P. G., & Jang, Y. (2019). Interspecific variation in seasonal migration and brumation behavior in two closely related species of treefrogs. *Frontiers in Ecology and Evolution*, 7, 55.

Borzée, A., Kim, J. Y., Da Cunha, M. A. M., Lee, D., Sin, E., Oh, S., ... Jang, Y. (2016). Temporal and spatial differentiation in microhabitat use: Implications for reproductive isolation and ecological niche specification. *Integrative Zoology*, 11, 375-387.

Garrido-Priego, M., Monge-Velázquez, M., Whitworth, A., & Gomez-Mestre, I. (2024). Home range and notes about social interactions in the poison frog *Phyllobates vittatus* (Anura: Dendrobatidae). *Evolutionary Ecology*, 1-12.

Gourret, A., Alford, R., & Schwarzkopf, L. (2011). Very small, light dipole harmonic tags for tracking small animals. *Herpetological Review*, 42, 522-525.

Kim E., Cahyana A. N., Jang Y., & Borzée A. (2019). Breeding range variation between Korean hylids (*Dryophytes* sp.). *Journal of Asia-Pacific Biodiversity*, 12, 135-138.

Kok, P. J. R., van Doorn, L., & Dezfoulian, R. (2019). Predation by non-bioluminescent firefly larvae on a tepui-summit endemic toad. *Current Biology*, 29, R1170–R1171.

Pašukonis, A., Loretto, M.-C., Landler, L., Ringler, M., & Hödl, W. Homing trajectories and initial orientation in a Neotropical territorial frog, *Allobates femoralis* (Dendrobatidae). (2014). *Frontiers in Zoology*, 11, 1-9.

Pašukonis, A., Warrington, I., Ringler, M., & Hödl, W. (2014). Poison frogs rely on experience to find the way home in the rainforest. *Biology Lettters*, 10, 17-20.

Pašukonis, A., Loretto, M. C., & Hödl, W. (2018). Map-like navigation from distances exceeding routine movements in the three-striped poison frog (*Ameerega trivittata*). *Journal of Experimental Biology*, 221(2), jeb169714.

Roznik, E. A., Alford, R. A. (2015) Seasonal Ecology and Behavior of an Endangered Rainforest Frog (*Litoria rheocola*) Threatened by Disease. PLoS ONE 10(5): e0127851.
